# A standardized protocol and basic ecological findings to include charophytes in functional trait studies

**DOI:** 10.1093/aob/mcag081

**Published:** 2026-04-16

**Authors:** Alice Dalla Vecchia, Rossano Bolpagni, Mattia M Azzella

**Affiliations:** Department of Chemistry, Life Sciences and Environmental Sustainability, University of Parma, 43124 Parma, Italy; Department of Chemistry, Life Sciences and Environmental Sustainability, University of Parma, 43124 Parma, Italy; Department of Planning, Design, Technology of Architecture, University of Rome La Sapienza, 00196 Rome, Italy

**Keywords:** Characeae, *Chara* spp, *Nitella* spp, *Nitellopsis obtusa*, leaf traits, functional niche, freshwater ecosystems, multidimensional macrophyte comparison, functional strategies

## Abstract

**Background and Scope:**

Charophytes comprise key benthic macroalgae that play multiple ecological roles in freshwater. Despite this, they are excluded from community-level functional trait research, leading to an underestimation of functional diversity of charophyte-dominated ecosystems. This study aims to develop a standardized protocol for measuring leaf functional traits for charophytes, and to provide a first functional comparison between charophytes and common vascular macrophyte life forms.

**Methods:**

Functional units were identified that perform the leaf function and reflect the modular growth of charophytes (whorls, branchlets and internodes). Leaf (branchlet) area, specific leaf area and leaf dry matter content, along with chlorophyll, carotenoid, phosphorus, nitrogen and carbon contents were measured on nine charophyte species. The protocol was validated by comparing traits between charophytes and five macrophyte groups (lemnids, nymphaeids, submerged-broad-leaf, submerged-narrow-leaf and utricularids), using a multidimensional niche approach.

**Key Results:**

Trait measurements were comparable between charophytes and vascular macrophytes. Charophytes’ functional traits showed alignment with ecological expectations, such as increased specific leaf area and reduced dry matter content along the expected growth depth. Overall, charophytes showed high intraspecific variability, attributable to their ability to grow across wide gradients of depth and light. Charophyte functional niche overlapped mostly with submerged narrow-leaved plants but maintained >40 % niche uniqueness.

**Conclusions:**

Charophytes expand the functional space occupied by macrophytes, contributing to understanding the overall functional diversity of aquatic plants. Following research should apply the protocol to all genera of the family Characeae and investigate the environmental drivers of trait variability.

## INTRODUCTION

Charophytes are benthic macroalgae (family Characeae) that play important ecological roles (Schneider *et al.*, 2015*a*; Schubert *et al.*, 2024) and have a complex morphology, resembling submerged vascular plants (Domozych *et al.*, 2016). Their relevance is highlighted by the definition of a habitat of European interest, the ‘Hard oligo-mesotrophic waters with benthic vegetation of *Chara* spp.’ (Habitat Directive 92/43/EEC, code 3140) (Dalla Vecchia *et al.*, 2025*a*).

Functional ecology of aquatic plant communities is a rapidly growing field of research (Dalla Vecchia *et al.*, 2020), but studies mainly focus on vascular species, excluding charophytes from comprehensive functional comparisons (e.g. Cavalli *et al.*, 2014; Fu *et al.*, 2014). Many studies considered traits of charophytes, showing morphological adaptations of charophytes to ecological gradients (Asaeda *et al.*, 2007; Brzozowski and Pełechaty, 2020) or changes in nutrients and temperature (Puche *et al.*, 2018; Choudhury *et al.*, 2019). However, they were mostly charophyte-specific and would not allow a direct comparison with vascular macrophytes (Schneider *et al.*, 2006, 2016; Baastrup-Spohr *et al.*, 2015; Pukacz *et al.*, 2024).

Functional trait protocols have been developed for vascular species (Cornelissen *et al.*, 2003; Pérez-Harguindeguy *et al.*, 2013). Charophytes are non-vascular species, therefore they do not have leaves that can be measured (Schubert *et al.*, 2024). We claim that the development of a standardized protocol for the measurement of charophyte traits would ultimately make wide-spectrum comparisons with vascular plants possible.

Here, we develop a standard protocol to measure the equivalent of leaf functional traits for charophytes, based on the concept of functional units, i.e. structural units that perform the function of leaves (Bolpagni *et al.*, 2024). We then validate the method by comparing key traits with five common vascular macrophyte groups (lemnids, nymphaeids, submerged broad-leaf species, submerged narrow-leaf species and utricularids), proposing a first multidimensional functional comparison.

## MATERIALS AND METHODS

### Study area and sampling design

**Fig. 1.**
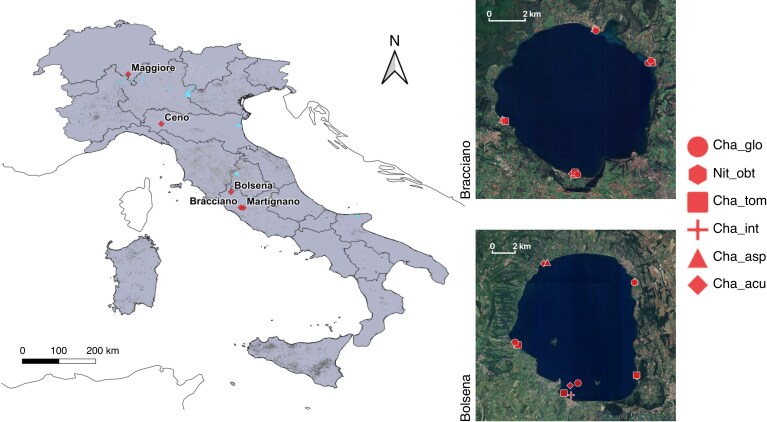
Map of the study sites, with examples of sampled species arrangement within lakes.

The sampling of charophytes was conducted in northern and central Italy and southern Switzerland, specifically in three deep volcanic lakes (Bracciano, Martignano and Bolsena), one subalpine lake (Maggiore) and one stream (Ceno, Fig. 1).

The design of a protocol for the measure of functional traits of charophytes was developed on nine species: *Chara aculeolata*, *C. aspera*, *C. globularis*, *C. papillosa*, *C. tomentosa*, *C. vulgaris*, *Nitella flexilis*, *Nitella hyalina* and *Nitellopsis obtusa*. These species were common in the study area, and they occupy different water depths, allowing us to cover a considerable range of charophyte variation. In general, we expect to find *C. aspera*, *C. papillosa*, *C. vulgaris* and *Nitella hyalina* in shallow water (0–3 m), *C. aculeolata*, *C. tomentosa* and *C. globularis* at intermediate depth (3–6 m), and *Nitellopsis obtusa* and *Nitella flexilis* in deeper water (Azzella *et al.*, 2017). For each species, we sampled from one to seven populations – independent stands at least 500 m apart from each other – depending on the abundance of the species in the study sites (Supplementary Data Table SI1).

### Functional trait protocol for charophytes

**Fig. 2.**
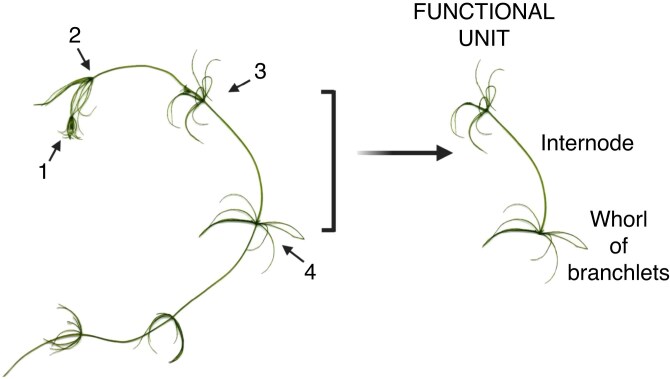
Determination of the functional unit composed of the third and fourth whorl of branchlets, including the internode. A sample of *Chara globularis* is displayed.

To measure leaf functional traits in charophytes we needed to define a leaf-like structure and take into consideration the high morphological and biochemical plasticity of charophytes, especially in relation to water depth and light availability (e.g. Schneider *et al.*, 2006, 2015*b*, 2016; Asaeda *et al.*, 2007; Brzozowski and Pełechaty, 2020). Therefore, we identified a functional unit that would represent the ‘leaf’ (see Bolpagni *et al.*, 2024), composed of two whorls (and their branchlets) and one internode (Fig. 2). This reflects the modular growth of charophytes (whorls + internode), and the second whorl was included to avoid loss of cellular content when severing the functional unit. To minimize trait variability due to the different developmental stages of the functional units, we always selected the third and fourth whorl from the apex (head) of the shoot, with the corresponding internode (Fig. 2).

We measured the following ‘leaf’ (functional unit) traits: area (LA); fresh and dry weight (LFW and LDW); dry matter content (LDMC); specific area (SLA); chlorophyll-*a* (Chl-*a*); chlorophyll-*b* (Chl-*b*); carotenoid (Car) content; chlorophyll-*a* to chlorophyll-*b* ratio (Chl-*a*/Chl-*b*); chlorophyll to carotenoid ratio (Chl/Car); and phosphorus (P), nitrogen (N) and carbon (C) contents. For each population, we measured ten replicates for LA, LFW, LDW, LDMC and SLA and five replicates for pigments. The dried functional units on which dry weight was measured were pooled for each population, and used to obtain one measure of P, N and C contents. We chose these traits as they are among the most common in macrophyte literature (Dalla Vecchia *et al.*, 2020) and have already been used for multiple species comparisons (Bolpagni *et al.*, 2024; Dalla Vecchia *et al.*, 2024, 2025*b*).

Once identified, the functional units were rinsed and cleaned with a brush from dirt, epiphytes or secondary encrustations. The carbonate encrustations were not removed. They are the result of inorganic carbon accumulation processes and can have a significant influence on charophyte biomass (Strzałek *et al.*, 2024). Oogones and antheridia were removed and not analysed, while stipulodes, bract and spine cells were included in the analysis. Functional units were weighed with a precision scale (0.0001 g) and scanned with a portable scanner at 600 dpi for the determination of fresh weight and area. Area was calculated with the software ImageJ (Schneider *et al.*, 2012) as the projected area of the scanned units. It was not possible to measure the area of *Nitella hyalina* due to the globular structure of its branchlets, which would be damaged in the process. Functional units were then dried for 48 h at 60 °C to determine dry weight. LDMC was calculated as the ratio between dry and fresh weight, and SLA as the ratio between area and dry weight (Pérez-Harguindeguy *et al.*, 2013). Pigments were determined spectrophotometrically after extraction of fresh functional units in 80 % acetone (Wellburn, 1994). Phosphorus was extracted from an incinerated subsample with HCl and determined spectrophotometrically (Aspila *et al.*, 1976). We determined N and C by combustion analysis with an elemental analyser (Thermo Flash EA 1112).

To validate the dataset of charophyte functional traits, we compared it with trait data of five other macrophyte groups following the classification by Hutchinson (1975): lemnids, nymphaeids, submerged broad-leaf (representing magnopotamids), submerged narrow-leaf (including parvopotamids, ceratophyllids, myriophyllids and *Stuckenia pectinata*) and utricularids. The comparison dataset included traits measured on 729 individuals (Supplementary Data SI2) (Bolpagni *et al.*, 2024; Villa *et al.*, 2025; Dalla Vecchia *et al.*, 2025*b*).

### Statistical analyses

All analyses were carried out in R (R Core Team, 2023). To test for differences in functional traits we used ANOVA or the Kruskal–Wallis test, followed by the Tukey or Dunn post hoc test, respectively (significance level set at 0.05).

To determine the functional space occupied by charophytes within macrophytes, we used a multidimensional niche approach based on probabilistic hypervolumes (Laini *et al.*, 2023), following the methodology described in Dalla Vecchia *et al.* (2024, 2025*b*). To compare niches, we used relative niche size, indicating the extent of the functional space occupied by each group; relative niche uniqueness, indicating the proportion of a group’s niche not shared with other groups; and niche intersection, indicating the proportion of the total volume shared by a pair of groups, i.e. the overlap between groups. Functional traits with Pearson’s correlation coefficient >|0.6| were omitted from the analysis, and the remaining traits were synthesized using PCA ordination. The species *Nitella hyalina* was excluded from this analysis as we did not have data for leaf area.

## RESULTS

### Functional traits of charophytes and protocol validation

**Fig. 3.**
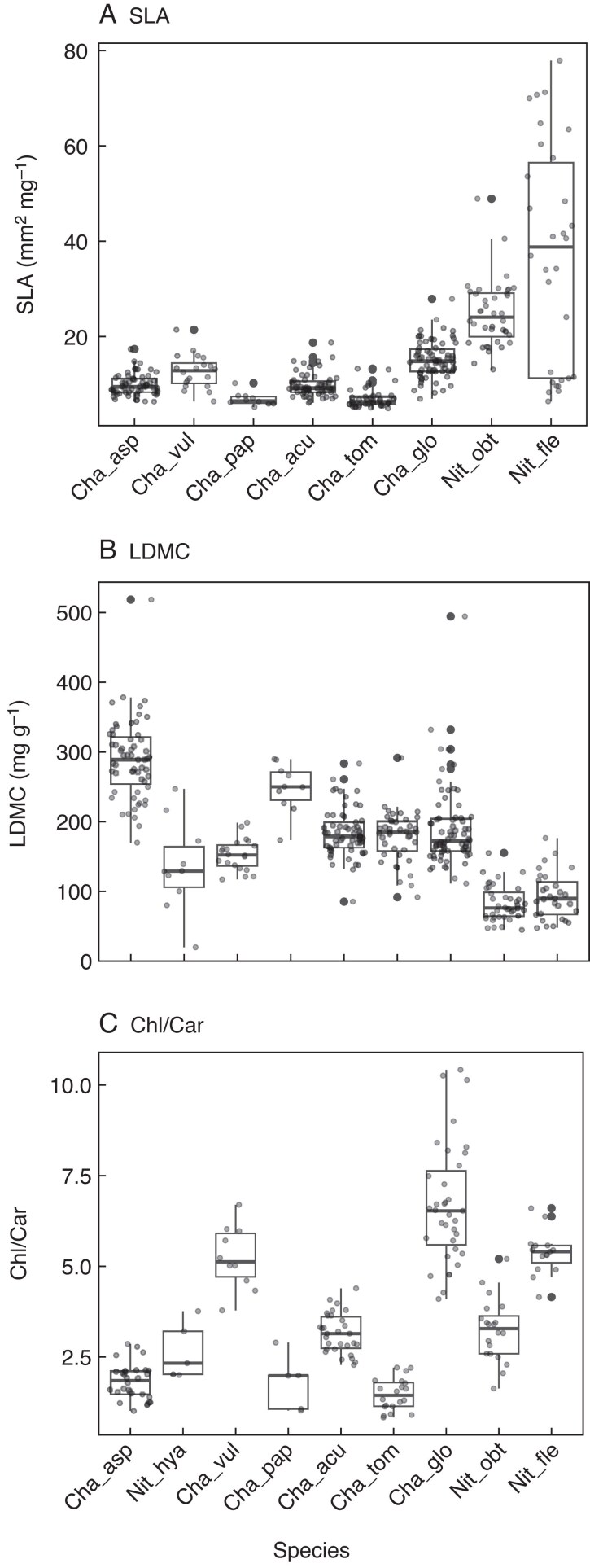
Distribution of SLA, LDMC and Chl/Car for the charophyte species analysed, ordered by increasing expected water depth from shallower to deeper. Cha_asp, *Chara aspera*; Nit_hya, *Nitella hyalina*; Cha_vul, *C. vulgaris*; Cha_pap, *C. papillosa*; Cha_acu, *C. aculeolata*; Cha_tom, *C. tomentosa*; Cha_glo, *C. globularis*; Nit_obt, *Nitellopsis obtusa*; Nit_fle, *Nitella flexilis*.

Charophyte SLA, LDMC and Chl/Car ratio reflected the expected water depth generally occupied by the species (Fig. 3). SLA was significantly higher in *Nitellopsis obtusa*, *Nitella flexilis* and *C. globularis*, expected to be found in deeper water, while we observed an almost opposite trend for LDMC (Fig. 3, Supplementary Data SI3). The Chl/Car ratio also partly reflected this gradient, as *C. globularis* and *Nitella flexilis*, which grow in deeper water, had the highest ratio (Fig. 3C, Supplementary Data SI3).

**Fig. 4.**
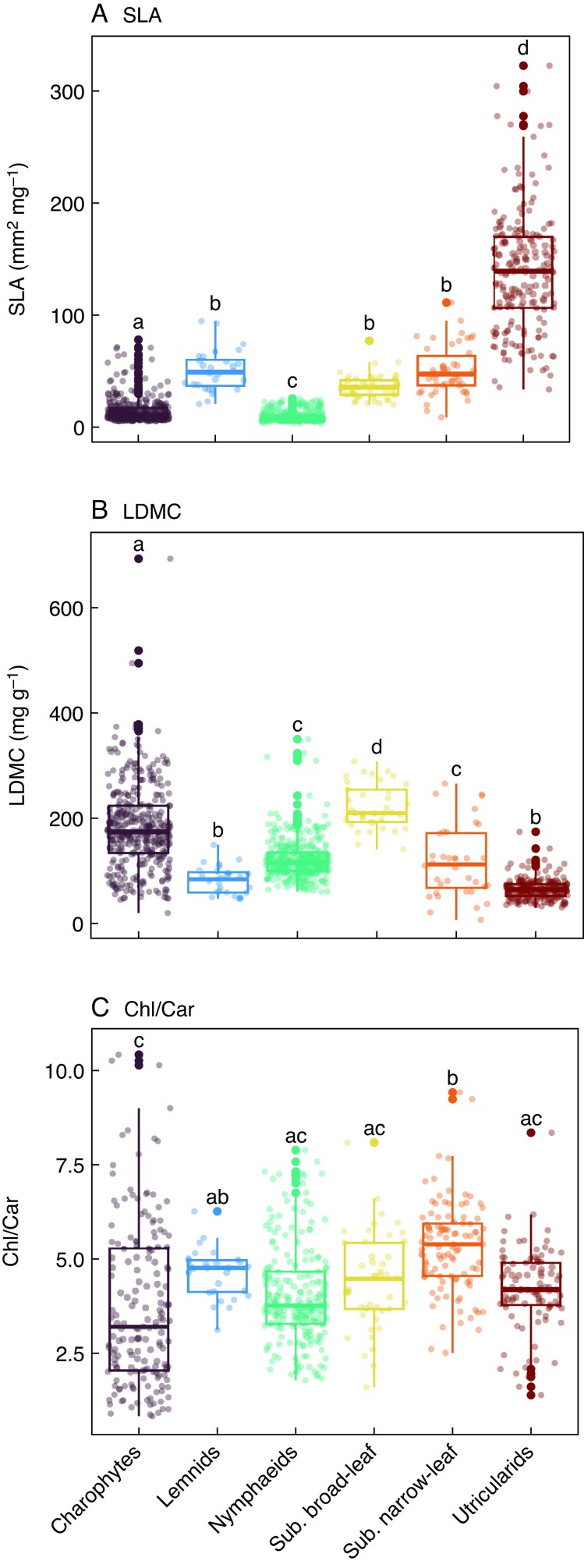
Distribution of SLA, LDMC and Chl/Car for the six macrophyte groups analysed. Different letters on the boxplots indicate significant differences between pairs of macrophyte groups.

Overall, functional trait values of charophytes largely overlapped with those of other macrophyte groups (Supplementary Data SI4), but they were distinguished by a significantly lower SLA (Fig. 4A, Supplementary Data SI4), Chl-*a*, N and C compared with most other groups (Supplementary Data SI5). Moreover, charophytes had the highest coefficient of variation for SLA, Chl/Car ratio and N content compared with other macrophyte groups (77.6, 57.1 and 53.6 % respectively; Supplementary Data SI4, Fig. 4A–C). Also, charophytes showed the highest range of variation for LDMC and Chl/Car ratio (Fig. 4B, C).

### Functional niche of charophytes

**Fig. 5.**
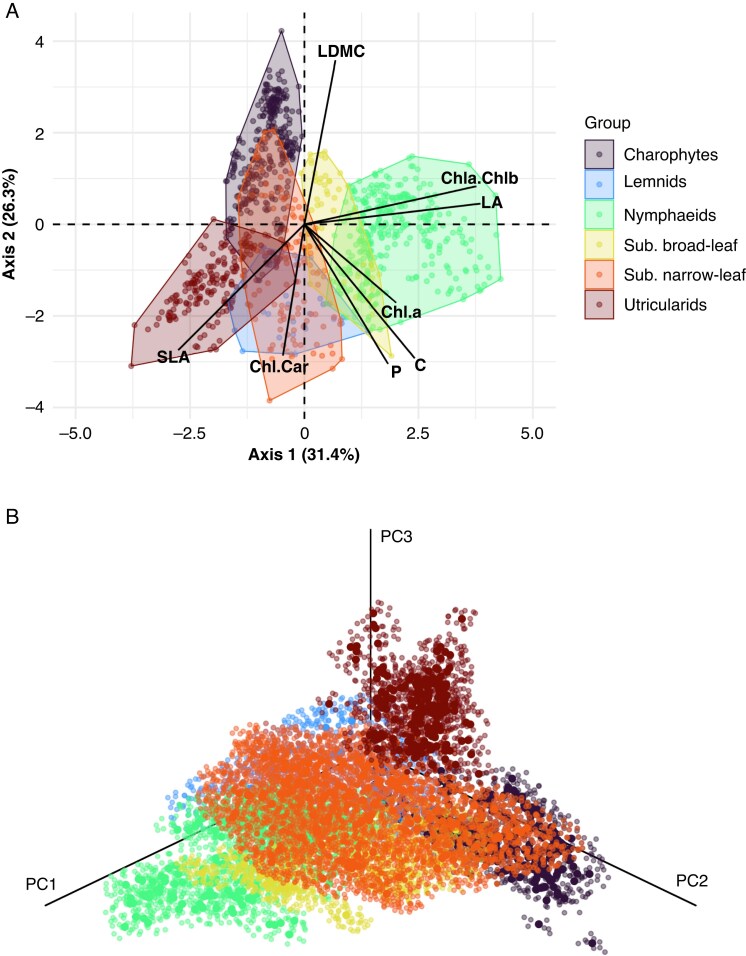
(A) Biplot of the first two PCA axes (variance explained in brackets). For trait abbreviations refer to the section Functional traits protocol for charophytes in the Results section. (B) Three-dimensional representation of functional niche hypervolumes. Solid points represent data points, while semitransparent points represent the estimated points by the hypervolume algorithm. See Supplementary Data SI9 for the interactive version of the 3D plot.

A PCA ordination was performed on eight weakly correlated traits (Table 1, Supplementary Data SI6), and the first three PCA axes together explained 74 % of the variance (Fig. 5A, Supplementary Data SI7).

**Table 1.** Variance explained (VE) for the first three principal components (PC1–PC3) and relative loadings of traits. For trait abbreviations, refer to the section Functional traits protocol for charophytes in the Results section.

	PC1	PC2	PC3
**VE**	**0**.**3139**	**0**.**2631**	**0**.**1592**
LA	0.5416	0.0634	0.1829
LDMC	0.0963	0.5051	−0.3940
SLA	−0.3874	−0.3859	0.2803
Chl-*a*	0.2803	−0.2381	−0.5233
Chl-*a*/Chl-*b*	0.5293	0.1167	0.1641
Chl/Car	−0.0658	−0.4028	−0.5449
P	0.2623	−0.4364	−0.1577
C	0.3397	−0.4110	0.3321

**Fig. 6.**
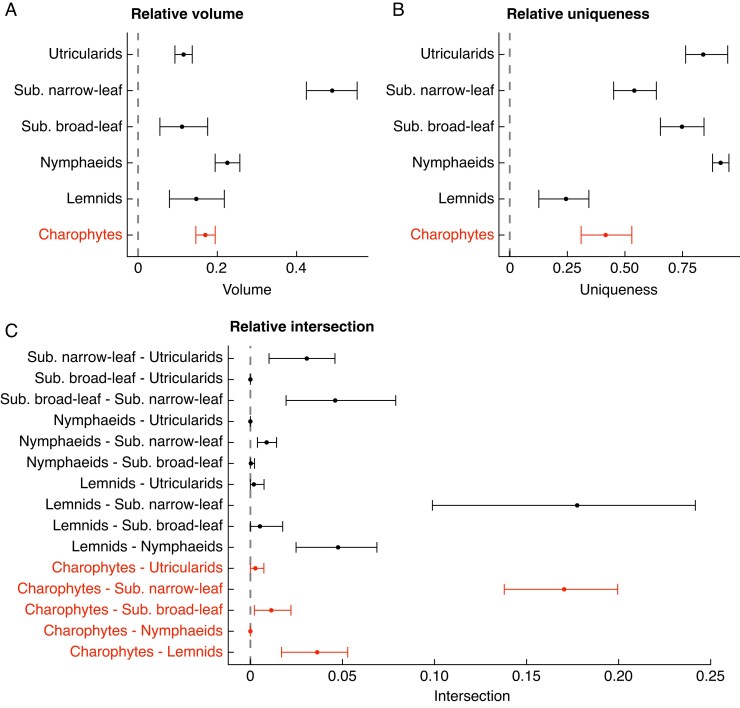
95 % credible intervals for relative functional niche volume (A), uniqueness (B) and intersection (C). Niche volume and intersection are relative to the union of all groups’ volumes, while uniqueness is relative to each group’s volume. Overlapping intervals indicate no significant difference between groups. Intersecting intervals including zero indicate no significant overlap between groups.

Charophytes had a mean relative niche volume of 16.9 %, significantly lower than submerged-narrow-leaf species and higher than utricularids (Fig. 6A, Supplementary Data SI8). The average niche uniqueness of charophytes was 41.7 %, comparable to that of lemnids and submerged narrow-leaf species, and significantly lower than nymphaeids, submerged broad-leaf species and utricularids (Fig. 6B, Supplementary Data SI8). Charophytes’ niche overlapped mainly with submerged narrow-leaf species (17.1 % of the union of all niches), and partly with lemnids and submerged broad-leaf species (Fig. 6C, Supplementary Data SI8 and SI9).

## DISCUSSION

The functional traits measured provide evidence that charophytes follow the response patterns observed for vascular aquatic plants. Indeed, water depth determines steep gradients in light availability, temperature and oxygen, as well as the influence of sediment metabolism (Huang *et al.*, 2019; Dalla Vecchia and Bolpagni, 2022). Generally, in submerged aquatic plants SLA tends to increase at greater water depth, while LDMC tends to decrease (Gao *et al.*, 2021), and the proportion of carotenoids is also modulated in leaves in response to light availability, with light-exposed leaves containing a higher proportion of carotenoids (thus lower Chl/Car ratio), and vice versa for shade-adapted leaves (Lichtenthaler, 2007; Schneider *et al.*, 2015*b*). We found that the investigated charophytes follow these general patterns, suggesting that commonly studied leaf traits can reasonably apply to non-vascular charophytes. Besides, we believe that the high trait variability observed reflects the adaptation to a wide environmental range, and it is not due to methodological shortcomings, as charophyte species can survive in very wide water depth ranges and some species tolerate brackish or hypersaline conditions as well (Schwarz *et al.*, 2002; Schneider *et al.*, 2015*a*). Moreover, enlarging the analysis to include more structural traits, like shoot diameter, could help explain the patterns observed in the variation of the traits measured in this study (e.g. SLA).

The functional niche of charophytes partially overlapped with submerged narrow-leaf species, while still presenting >40 % of niche uniqueness. No or very little overlap was observed with nymphaeids, utricularids and lemnids, which was expected as these groups differ in the type of aquatic environment they occupy and in their resource acquisition strategies (Dalla Vecchia *et al.*, 2024, 2025*b*), and it further confirms the applicability and comparability of the developed protocol.

Part of charophytes’ uniqueness was due to a lower contents of Chl-*a* and nutrients (especially N and C). The low nutrient and chlorophyll contents may reflect the precipitation of CaCO_3_ due to photosynthesis, which contributes up to >65 % to the dry weight of charophytes (Brzozowski and Pełechaty, 2025), but also measuring ash content could help explain this result. Their relatively limited niche size, especially compared with submerged narrow-leaf species, suggests that charophytes are very specialized (Devictor *et al.*, 2010), and can thus show high performance given the right conditions.

Investigating the drivers of the high variability observed in some traits, and including physiological traits that are more informative of plant performance, would allow a better understanding of charophytes’ adaptation limits. This would be a key step to determine how charophytes could survive in changing conditions, as multiple stressors (eutrophication, competition from vascular species and impact of herbivores) are threatening this important ecosystem component (Alirangues-Nuñez *et al.*, 2023). The ecological role of charophytes is well documented, highlighting their ability to influence nutrient cycles and to support biotic communities in the different stages of the growing season (Hargeby, 1990; Hilt *et al.*, 2025). Therefore, future studies should consider traits like biomass density and phenology, including length of the growing season, coupled with local environmental conditions, to better determine their functional role in the environment. Indeed, the chosen species belong to only three of the seven known genera of charophytes. Applying this protocol to other genera of the family Characeae (*Lamprothamnium*, *Lychnothamnus*, *Tolypella* and *Sphaerochara*) would be a key step to ensure a more complete understanding of functional diversity of charophyte-dominated aquatic habitats.

## Supplementary Material

mcag081_Supplementary_Data

## Data Availability

The charophyte functional trait dataset used for this research is available on the Zenodo.org repository at: https://doi.org/10.5281/zenodo.19615625.

## SUPPLEMENTARY DATA

Supplementary data are available at *Annals of Botany* online and consist of the following. SI1: number of populations sampled per species and study site. SI2: species composition of the comparison macrophyte dataset. SI3: post hoc tests of trait comparison among charophyte species. SI4: mean, standard deviation and coefficient of variation of traits for the six macrophyte groups investigated. SI5: post hoc tests for trait comparison among macrophyte groups. SI6: correlation matrix of leaf functional traits of all macrophyte groups. SI7: complete PCA biplots. SI8: credible intervals for relative niche volume, uniqueness and intersection of the six macrophyte groups investigated. SI9: interactive 3-D representation of the hypervolumes.

## References

[mcag081-B1] Alirangues-Nuñez MM, Hussner A, Mauersberger R, et al 2023. Periphyton and benthivorous fish affect charophyte abundance and indicate hidden nutrient loading in oligo- and mesotrophic temperate hardwater lakes. Freshwater Biology 68: 312–324. doi:10.1111/fwb.14026

[mcag081-B2] Asaeda T, Rajapakse L, Sanderson B. 2007. Morphological and reproductive acclimations to growth of two charophyte species in shallow and deep water. Aquatic Botany 86: 393–401. doi:10.1016/J.AQUABOT.2007.01.010

[mcag081-B3] Aspila KI, Agemian H, Chau ASY. 1976. A semi-automated method for the determination of inorganic, organic and total phosphate in sediments. The Analyst 101: 187–197. doi:10.1039/AN9760100187 1259177

[mcag081-B4] Azzella MM, Bresciani M, Nizzoli D, Bolpagni R. 2017. Aquatic vegetation in deep lakes: macrophyte co-occurrence patterns and environmental determinants. Journal of Limnology 76: 97–108. doi:10.4081/jlimnol.2017.1687

[mcag081-B5] Baastrup-Spohr L, Iversen LL, Borum J, Sand-Jensen K. 2015. Niche specialization and functional traits regulate the rarity of charophytes in the Nordic countries. Aquatic Conservation: Marine and Freshwater Ecosystems 25: 609–621. doi:10.1002/aqc.2544

[mcag081-B6] Bolpagni R, Adamec L, Dalla Vecchia A. 2024. Measuring standardized functional leaf traits of aquatic carnivorous plants – challenges and opportunities. Perspectives in Plant Ecology, Evolution and Systematics 65:125826. doi:10.1016/j.ppees.2024.125826

[mcag081-B7] Brzozowski M, Pełechaty M. 2020. Broad morphological and reproductive variability of the endangered macroalga *Lychnothamnus barbatus* in the depth gradient. Aquatic Botany 165:103239. doi:10.1016/j.aquabot.2020.103239

[mcag081-B8] Brzozowski M, Pełechaty M. 2025. Calcium carbonate content for two morphotypes of an indicator charophyte species across a depth and light gradient in a mesotrophic lake. Global Ecology and Conservation 59:e03546. doi:10.1016/j.gecco.2025.e03546

[mcag081-B9] Cavalli G, Baattrup-Pedersen A, Riis T. 2014. The role of species functional traits in distributional patterns of lowland stream vegetation. Freshwater Science 33: 1074–1085. doi:10.1086/678048

[mcag081-B10] Choudhury MI, Urrutia-Cordero P, Zhang H, Ekvall MK, Medeiros LR, Hansson LA. 2019. Charophytes collapse beyond a critical warming and brownification threshold in shallow lake systems. The Science of the Total Environment 661: 148–154. doi:10.1016/j.scitotenv.2019.01.177 30669047

[mcag081-B11] Cornelissen JHC, Lavorel S, Garnier E, et al 2003. A handbook of protocols for standardised and easy measurement of plant functional traits worldwide. Australian Journal of Botany 51: 335–380. doi:10.1071/BT02124

[mcag081-B12] Dalla Vecchia A, Adamec L, Bolpagni R. 2025b. Aquatic carnivorous plants fill gaps in the functional niches of macrophytes: intra-species variability and group strategies. Perspectives in Plant Ecology, Evolution and Systematics 67:125871. doi:10.1016/J.PPEES.2025.125871

[mcag081-B13] Dalla Vecchia A, Bolpagni R. 2022. The importance of being petioled: leaf traits and resource-use strategies in *Nuphar lutea*. Hydrobiologia 849: 3801–3812. doi:10.1007/s10750-022-04803-1

[mcag081-B14] Dalla Vecchia A, Bolpagni R, Laini A, Nizzoli D, Azzella MM, Wilkes M. 2025a. Spatial relationships between macrophyte assemblages, water and sediment features in deep lakes. Frontiers in Environmental Science 13:1614281. doi:10.3389/fenvs.2025.1614281

[mcag081-B15] Dalla Vecchia A, Castellani MB, Azzella MM, Bolpagni R. 2024. Ecological and functional niches comparison reveals differentiated resource-use strategies and ecological thresholds in four key floating-leaved macrophytes. Limnology and Oceanography 69: 1707–1719. doi:10.1002/LNO.12611

[mcag081-B16] Dalla Vecchia A, Villa P, Bolpagni R. 2020. Functional traits in macrophyte studies: current trends and future research agenda. Aquatic Botany 167:103290. doi:10.1016/j.aquabot.2020.103290

[mcag081-B17] Devictor V, Clavel J, Julliard R, et al 2010. Defining and measuring ecological specialization. Journal of Applied Ecology 47: 15–25. doi:10.1111/J.1365-2664.2009.01744.X

[mcag081-B18] Domozych DS, Popper ZA, Sørensen I. 2016. Charophytes: evolutionary giants and emerging model organisms. Frontiers in Plant Science 7:1470. doi:10.3389/FPLS.2016.01470 27777578 PMC5056234

[mcag081-B19] Fu H, Zhong J, Yuan G, Ni L, Xie P, Cao T. 2014. Functional traits composition predict macrophytes community productivity along a water depth gradient in a freshwater lake. Ecology and Evolution 4: 1516–1523. doi:10.1002/ece3.1022 24967072 PMC4063455

[mcag081-B20] Gao Y, Wang L, Hu X, et al 2021. Rapid adaptive responses of rosette-type macrophyte *Vallisneria natans* juveniles to varying water depths: the role of leaf trait plasticity. Ecology and Evolution 11: 14268–14281. doi:10.1002/ECE3.8142 34707853 PMC8525151

[mcag081-B21] Hargeby A . 1990. Macrophyte associated invertebrates and the effects of habitat permanence. Oikos 57: 338–346. doi:10.2307/3565963

[mcag081-B22] Hilt S, van de Weyer K, Meis S, et al 2025. Facilitation of lake eutrophication by altered feedback loops between submerged macrophyte vegetation and phosphorus retention. Freshwater Biology 70:e70051. doi:10.1111/fwb.70051

[mcag081-B23] Huang Y, Yang C, Wen C, Wen G. 2019. S-type dissolved oxygen distribution along water depth in a canyon-shaped and algae blooming water source reservoir: reasons and control. International Journal of Environmental Research and Public Health 16:987. doi:10.3390/ijerph16060987 30893863 PMC6466274

[mcag081-B24] Hutchinson GE . 1975. A treatise on limnology, Vol. 3. Limnological botany. New York: John Wiley.

[mcag081-B25] Laini A, Datry T, Blonder BW. 2023. N-dimensional hypervolumes in trait-based ecology: does occupancy rate matter? Functional Ecology 37: 1802–1814. doi:10.1111/1365-2435.14344

[mcag081-B26] Lichtenthaler HK . 2007. Biosynthesis, accumulation and emission of carotenoids, α-tocopherol, plastoquinone, and isoprene in leaves under high photosynthetic irradiance. Photosynthesis Research 92: 163–179. doi:10.1007/S11120-007-9204-Y 17634750

[mcag081-B27] Pérez-Harguindeguy N, Díaz S, Garnier E, et al 2013. New handbook for standardised measurement of plant functional traits worldwide. Australian Journal of Botany 61: 167–234. doi:10.1071/BT12225

[mcag081-B28] Puche E, Sánchez-Carrillo S, Álvarez-Cobelas M, Pukacz A, Rodrigo MA, Rojo C. 2018. Effects of overabundant nitrate and warmer temperatures on charophytes: the roles of plasticity and local adaptation. Aquatic Botany 146: 15–22. doi:10.1016/j.aquabot.2018.01.003

[mcag081-B29] Pukacz A, Asaeda T, Blindow I, et al 2024. Ecology of charophytes: niche dimensions of pioneers and habitat engineers. In: Schubert H, Blindow I, Nat E, et al Charophytes of Europe. Cham: Springer, 125–152. doi:10.1007/978-3-031-31898-6_7

[mcag081-B30] R Core Team . 2023. R: a language and environment for statistical computing. Vienna, Austria: R Foundation for Statistical Computing. https://www.R-project.org/ (5 October 2025, date last accessed).

[mcag081-B31] Schneider SC, García A, Martín-Closas C, Chivas AR. 2015a. The role of charophytes (Charales) in past and present environments: an overview. Aquatic Botany 120: 2–6. doi:10.1016/J.AQUABOT.2014.10.001

[mcag081-B32] Schneider SC, Nowak P, Von Ammon U, Ballot A. 2016. Species differentiation in the genus Chara (Charophyceae): considerable phenotypic plasticity occurs within homogenous genetic groups. European Journal of Phycology 51: 282–293. doi:10.1080/09670262.2016.1147085

[mcag081-B33] Schneider SC, Pichler DE, Andersen T, Melzer A. 2015b. Light acclimation in submerged macrophytes: the roles of plant elongation, pigmentation and branch orientation differ among *Chara* species. Aquatic Botany 120: 121–128. doi:10.1016/J.AQUABOT.2014.05.002

[mcag081-B34] Schneider CA, Rasband WS, Eliceiri KW. 2012. NIH Image to ImageJ: 25 years of image analysis. Nature Methods 9: 671–675. doi:10.1038/nmeth.2089 22930834 PMC5554542

[mcag081-B35] Schneider S, Ziegler C, Melzer A. 2006. Growth towards light as an adaptation to high light conditions in *Chara* branches. New Phytologist 172: 83–91. doi:10.1111/J.1469-8137.2006.01812.X 16945091

[mcag081-B36] Schubert H, Blindow I, Nat E, et al 2024. Charophytes of Europe. Cham: Springer.

[mcag081-B37] Schwarz AM, de Winton M, Hawes I. 2002. Species-specific depth zonation in New Zealand charophytes as a function of light availability. Aquatic Botany 72: 209–217. doi:10.1016/S0304-3770(01)00201-7

[mcag081-B38] Strzałek M, Kufel L, Apolinarska K, et al 2024. Recycling and deposition of inorganic carbon from calcium carbonate encrustations of charophytes. Limnology and Oceanography 69: 279–289. doi:10.1002/lno.12479

[mcag081-B39] Villa P, Berton A, Bolpagni R, et al 2025. Exploring spectral and phylogenetic diversity links with functional structure of aquatic plant communities. Remote Sensing of Environment 318:114582. doi:10.1016/J.RSE.2024.114582

[mcag081-B40] Wellburn AR . 1994. The spectral determination of chlorophylls a and b, as well as total carotenoids, using various solvents with spectrophotometers of different resolution. Journal of Plant Physiology 144: 307–313. doi:10.1016/S0176-1617(11)81192-2

