# Supplementary material for: A standardized protocol and basic ecological findings to include charophytes in functional trait studies
Source: Ann Bot. 2026 Apr 16;138(2):307–14. doi: 10.1093/aob/mcag081 (PMC13454718; doi:10.1093/aob/mcag081)
Supplement: Supplementary file 1 [file mcag081_supplementary_data.zip › Supplementary_Data_Charophyte.docx]

Supplementary information for article:

**A standardized protocol and basic ecological findings to include charophytes in functional trait studies**

Alice Dalla Vecchia^1*†^, Rossano Bolpagni^1†^, Mattia M. Azzella^2^

^1^ Department of Chemistry, Life Sciences and Environmental Sustainability, University of Parma, Parma, Italy

^2^ Department of Planning, Design, Technology of Architecture, Land and Environment, University of Rome La Sapienza, Rome, Italy

*For correspondence. Email: [alice.dallavecchia@unipr.it](mailto:alice.dallavecchia@unipr.it)

^†^These authors contributed equally to this work and should be considered co-first authors

**SI1**: Number of populations sampled per species and study site, along with average coordinates of the study sites and total number of populations sampled per species.

| **Species** | **Bracciano**  42.1194; 12.2325 | **Martignano**  42.1121;  12.3163 | **Bolsena**  42.5896;  11.9447 | **Maggiore**  45.1594;  8.8317 | **Ceno**  44.6579;  9.8331 | **Total** |
| --- | --- | --- | --- | --- | --- | --- |
| *Chara globularis* | 3 | 1 | 3 |  |  | 7 |
| *Chara aculeolata* | 3 |  | 3 |  |  | 6 |
| *Chara aspera* | 3 |  | 3 |  |  | 6 |
| *Chara tomentosa* | 1 |  | 3 |  |  | 4 |
| *Nitellopsis obtusa* | 1 | 3 |  |  |  | 4 |
| *Nitella flexilis* |  |  |  | 3 |  | 3 |
| *Chara vulgaris* |  |  |  |  | 2 | 2 |
| *Chara papillosa* |  |  | 1 |  |  | 1 |
| *Nitella hyalina* |  | 1 |  |  |  | 1 |

**SI2:** Number of observations per species for each group of macrophytes included in this study. References to previous publications are reported in the main manuscript.

| **Species** | **Macrophyte group** | **N. observations** | **Previous publications** |
| --- | --- | --- | --- |
| *Chara aculeolata* | Charophytes | 60 |  |
| *Chara aspera* | Charophytes | 60 |  |
| *Chara globularis* | Charophytes | 70 |  |
| *Chara tomentosa* | Charophytes | 40 |  |
| *Chara papillosa* | Charophytes | 10 |  |
| *Chara vulgaris* | Charophytes | 20 |  |
| *Nitella flexilis* | Charophytes | 30 |  |
| *Nitella hyalina* | Charophytes | 10 |  |
| *Nitellopsis obtusa* | Charophytes | 40 |  |
| *Lemna gibba* | Lemnids | 5 | Bolpagni et al., 2024; Villa et al., 2025; Dalla Vecchia et al., 2025b |
| *Lemna minor* | Lemnids | 4 |  |
| *Lemna minuta* | Lemnids | 5 |  |
| *Lemna trisulca* | Lemnids | 5 |  |
| *Spirodela polyrhiza* | Lemnids | 5 |  |
| *Nelumbo nucifera* | Nymphaeids | 60 | Dalla Vecchia et al., 2024a-b; Bolpagni et al., 2024; Villa et al., 2025; Dalla Vecchia et al., 2025b |
| *Nuphar lutea* | Nymphaeids | 140 |  |
| *Nymphaea alba* | Nymphaeids | 81 |  |
| *Nymphoides peltata* | Nymphaeids | 60 |  |
| *Potamogeton lucens* | Sub.broad.leaf | 9 | Bolpagni et al., 2024; Villa et al., 2025; Dalla Vecchia et al., 2025b |
| *Potamogeton perfoliatus* | Sub.broad.leaf | 36 |  |
| *Ceratophyllum demersum* | Sub.narrow.leaf | 33 |  |
| *Elodea canadensis* | Sub.narrow.leaf | 3 |  |
| *Elodea nuttallii* | Sub.narrow.leaf | 3 |  |
| *Lagarosiphon major* | Sub.narrow.leaf | 18 |  |
| *Myriophyllum spicatum* | Sub.narrow.leaf | 38 |  |
| *Najas marina* | Sub.narrow.leaf | 12 |  |
| *Najas minor* | Sub.narrow.leaf | 6 |  |
| *Stuckenia pectinata* | Sub.narrow.leaf | 12 |  |
| *Aldrovanda vesiculosa* | Utricularids | 30 | Bolpagni et al., 2024; Dalla Vecchia et al., 2025b |
| *Utricularia australis* | Utricularids | 30 |  |
| *Utricularia bremii* | Utricularids | 30 |  |
| *Utricularia intermedia* | Utricularids | 20 |  |
| *Utricularia minor* | Utricularids | 20 |  |
| *Utricularia ochroleuca* | Utricularids | 20 |  |
| *Utricularia stygia* | Utricularids | 30 |  |
| *Utricularia vulgaris* | Utricularids | 10 |  |
| *Wolffia arrhiza* | Utricularids | 4 |  |

**SI3**: Results of the post hoc tests (Tukey – dark grey, or Dunn test – light grey) for the comparison of traits among charophyte species. Diff=mean difference between species, P-adj= adjusted p-value. Significance level set at p<0.05, significant differences are highlighted in bold. Only traits with at least one significant difference are shown. LA=Leaf Area, SLA=Specific Leaf Area (leaf area/leaf dry weight), LDMC=Leaf Dry Matter Content (leaf dry weight/leaf fresh weight), Chl-a=Chlorophyll-a, Car=Carotenoids, Chl-a/Chl-b=Chlorophyll-a to Chlorophyll-b ratio, Chl/Car=Chlorophylls to Carotenoids ratio, P=leaf Phosphorus content, N=leaf Nitrogen content, C=leaf Carbon content. Cha_acu=*Chara aculeolata*, Cha_asp=*C. aspera*, Cha_pap=*C. intermedia*, Cha_glo=*C. globularis*, Cha_tom=*C. tomentosa*, Cha_vul=*C. vulgaris*, Nit_hya=*Nitella hyalina*, Nit_obt=*Nitellopsis obtusa*.

|  | **LA**  [mm^2^] | | **SLA**  [mm^2^/mg] | | **LDMC**  [mg/g] | |
| --- | --- | --- | --- | --- | --- | --- |
| **Comparison** | Diff | P.adj | Diff | P.adj | Diff | P.adj |
| Nit_hya-Cha_asp | NA | NA | NA | NA | **-153.59** | **<0.0001** |
| Cha_vul-Cha_asp | **367.52** | **<0.0001** | 2.74 | 0.1568 | **-136.45** | **<0.0001** |
| Cha_pap-Cha_asp | 82.70 | 0.3264 | -3.02 | 0.1229 | -41.15 | 0.1554 |
| Cha_acu-Cha_asp | **513.05** | **<0.0001** | -0.08 | 1.0000 | **-104.86** | **<0.0001** |
| Cha_tom-Cha_asp | **382.62** | **<0.0001** | **-2.85** | **0.0016** | **-110.48** | **<0.0001** |
| Cha_glo-Cha_asp | **173.58** | **<0.0001** | **5.24** | **<0.0001** | **-98.95** | **<0.0001** |
| Nit_obt-Cha_asp | **474.45** | **<0.0001** | **14.88** | **<0.0001** | **-206.45** | **<0.0001** |
| Nit_fle-Cha_asp | 84.11 | 0.0221 | **27.13** | **<0.0001** | **-195.94** | **<0.0001** |
| Cha_vul-Nit_hya | NA | NA | NA | NA | 17.14 | 0.9867 |
| Cha_pap-Nit_hya | NA | NA | NA | NA | **112.44** | **<0.0001** |
| Cha_acu-Nit_hya | NA | NA | NA | NA | **48.73** | **0.0410** |
| Cha_tom-Nit_hya | NA | NA | NA | NA | 43.11 | 0.1427 |
| Cha_glo-Nit_hya | NA | NA | NA | NA | **54.64** | **0.0104** |
| Nit_obt-Nit_hya | NA | NA | NA | NA | **-52.86** | **0.0256** |
| Nit_fle-Nit_hya | NA | NA | NA | NA | -42.35 | 0.1937 |
| Cha_pap-Cha_vul | **-284.82** | **0.0084** | **-5.76** | **0.0029** | **95.30** | **<0.0001** |
| Cha_acu-Cha_vul | 145.53 | 0.8011 | -2.82 | 0.1367 | 31.59 | 0.1397 |
| Cha_tom-Cha_vul | 15.10 | 1.0000 | **-5.59** | **<0.0001** | 25.97 | 0.4614 |
| Cha_glo-Cha_vul | **-193.94** | **0.0085** | 2.51 | 0.4693 | **37.50** | **0.0287** |
| Nit_obt-Cha_vul | 106.93 | 1.0000 | **12.14** | **0.0008** | **-70.01** | **<0.0001** |
| Nit_fle-Cha_vul | **-283.40** | **0.0001** | 24.39 | 0.0553 | **-59.49** | **0.0002** |
| Cha_acu-Cha_pap | **430.35** | **<0.0001** | 2.94 | 0.1285 | **-63.71** | **0.0013** |
| Cha_tom-Cha_pap | **299.92** | **0.0084** | 0.17 | 0.8856 | **-69.33** | **0.0005** |
| Cha_glo-Cha_pap | 90.88 | 0.9239 | **8.26** | **<0.0001** | **-57.80** | **0.0049** |
| Nit_obt-Cha_pap | **391.75** | **0.0002** | **17.90** | **<0.0001** | **-165.30** | **<0.0001** |
| Nit_fle-Cha_pap | 1.41 | 0.9877 | **30.15** | **<0.0001** | **-154.79** | **<0.0001** |
| Cha_tom-Cha_acu | -130.43 | 0.1305 | **-2.77** | **0.0028** | -5.62 | 0.9995 |
| Cha_glo-Cha_acu | **-339.47** | **<0.0001** | **5.32** | **<0.0001** | 5.91 | 0.9980 |
| Nit_obt-Cha_acu | -38.60 | 1.0000 | **14.96** | **<0.0001** | **-101.59** | **<0.0001** |
| Nit_fle-Cha_acu | **-428.93** | **<0.0001** | **27.21** | **<0.0001** | **-91.08** | **<0.0001** |
| Cha_glo-Cha_tom | **-209.04** | **0.0017** | **8.09** | **<0.0001** | 11.53 | 0.9308 |
| Nit_obt-Cha_tom | 91.83 | 0.7679 | **17.73** | **<0.0001** | **-95.97** | **<0.0001** |
| Nit_fle-Cha_tom | **-298.50** | **<0.0001** | **29.98** | **<0.0001** | **-85.46** | **<0.0001** |
| Nit_obt-Cha_glo | **300.87** | **<0.0001** | **9.64** | **0.0041** | **-107.50** | **<0.0001** |
| Nit_fle-Cha_glo | -89.46 | 0.3375 | 21.89 | 0.3214 | **-96.99** | **<0.0001** |
| Nit_fle-Nit_obt | **-390.33** | **<0.0001** | 12.25 | 0.6337 | 10.52 | 0.988019 |
|  | **Chl-a**  [μg/g FW] | | **Car**  [μg/g FW] | | **Chl-a/Chl-b** | |
| **Comparison** | Diff | P.adj | Diff | P.adj | Diff | P.adj |
| Nit_hya-Cha_asp | 152.05 | 1.0000 | -59.77 | 1.0000 | -0.140 | 0.7928 |
| Cha_vul-Cha_asp | 71.56 | 1.0000 | -207.58 | 1.0000 | 0.067 | 0.9834 |
| Cha_pap-Cha_asp | -228.66 | 0.1025 | -166.34 | 0.2340 | 0.031 | 1.0000 |
| Cha_acu-Cha_asp | **-195.32** | **0.0002** | **-239.37** | **0.0006** | **0.240** | **<0.0001** |
| Cha_tom-Cha_asp | **-274.83** | **<0.0001** | **-189.60** | **<0.0001** | **0.178** | **0.0210** |
| Cha_glo-Cha_asp | 114.17 | 0.9650 | -218.23 | 0.1211 | **-0.390** | **<0.0001** |
| Nit_obt-Cha_asp | **-153.78** | **0.0442** | -220.01 | 0.5709 | **-0.294** | **<0.0001** |
| Nit_fle-Cha_asp | **471.69** | **0.0056** | **-119.25** | **0.0033** | 0.088 | 0.8274 |
| Cha_vul-Nit_hya | -80.49 | 1.0000 | -147.82 | 0.9261 | 0.207 | 0.4711 |
| Cha_pap-Nit_hya | -380.71 | 0.1666 | -106.57 | 0.2121 | 0.171 | 0.8508 |
| Cha_acu-Nit_hya | -347.37 | 0.0534 | **-179.60** | **0.0420** | **0.380** | **0.0007** |
| Cha_tom-Nit_hya | **-426.88** | **0.0030** | **-129.84** | **0.0033** | **0.318** | **0.0146** |
| Cha_glo-Nit_hya | -37.88 | 1.0000 | -158.47 | 1.0000 | -0.249 | 0.0943 |
| Nit_obt-Nit_hya | -305.83 | 0.2901 | -160.25 | 0.6099 | -0.154 | 0.7374 |
| Nit_fle-Nit_hya | 319.64 | 1.0000 | -59.49 | 1.0000 | 0.228 | 0.2561 |
| Cha_pap-Cha_vul | **-300.22** | **0.0243** | 41.24 | 0.0974 | -0.036 | 1.0000 |
| Cha_acu-Cha_vul | **-266.88** | **0.0003** | **-31.79** | **0.0019** | 0.173 | 0.1752 |
| Cha_tom-Cha_vul | **-346.39** | **<0.0001** | **17.98** | **<0.0001** | 0.111 | 0.8050 |
| Cha_glo-Cha_vul | 42.62 | 0.9793 | -10.65 | 1.0000 | **-0.456** | **<0.0001** |
| Nit_obt-Cha_vul | **-225.34** | **0.0134** | -12.43 | 0.2389 | **-0.361** | **<0.0001** |
| Nit_fle-Cha_vul | 400.14 | 1.0000 | 88.33 | 0.5557 | 0.021 | 1.0000 |
| Cha_acu-Cha_pap | 33.34 | 1.0000 | -73.03 | 1.0000 | 0.210 | 0.2805 |
| Cha_tom-Cha_pap | -46.17 | 1.0000 | -23.27 | 1.0000 | 0.147 | 0.7808 |
| Cha_glo-Cha_pap | **342.83** | **0.0051** | **-51.90** | **0.0023** | **-0.420** | **0.0001** |
| Nit_obt-Cha_pap | 74.88 | 1.0000 | -53.67 | 1.0000 | **-0.324** | **0.0115** |
| Nit_fle-Cha_pap | **700.36** | **<0.0001** | **47.09** | **0.0001** | 0.057 | 0.9995 |
| Cha_tom-Cha_acu | -79.51 | 0.9718 | 49.76 | 1.0000 | -0.062 | 0.9539 |
| Cha_glo-Cha_acu | **309.49** | **<0.0001** | **21.13** | **<0.0001** | **-0.630** | **<0.0001** |
| Nit_obt-Cha_acu | 41.54 | 1.0000 | 19.36 | 0.9563 | **-0.534** | **<0.0001** |
| Nit_fle-Cha_acu | **667.01** | **<0.0001** | **120.12** | **<0.0001** | -0.152 | 0.1626 |
| Cha_glo-Cha_tom | **389.00** | **<0.0001** | **-28.63** | **<0.0001** | **-0.567** | **<0.0001** |
| Nit_obt-Cha_tom | 121.05 | 0.2686 | -30.41 | 0.0814 | **-0.472** | **<0.0001** |
| Nit_fle-Cha_tom | **746.53** | **<0.0001** | **70.35** | **<0.0001** | -0.090 | 0.8708 |
| Nit_obt-Cha_glo | **-267.95** | **0.0001** | **-1.78** | **0.0002** | 0.096 | 0.6115 |
| Nit_fle-Cha_glo | 357.52 | 0.2802 | 98.98 | 0.9795 | **0.478** | **<0.0001** |
| Nit_fle-Nit_obt | **625.48** | **<0.0001** | **100.76** | **<0.0001** | **0.382** | **<0.0001** |

|  | **Chl/Car** | | **N**  [% DW] | | **C**  [% DW] | |
| --- | --- | --- | --- | --- | --- | --- |
| **Comparison** | Diff | P.adj | Diff | P.adj | Diff | P.adj |
| Nit_hya-Cha_asp | 0.84 | 1.0000 | 0.842 | 1.0000 | 2.12 | 0.9973 |
| Cha_vul-Cha_asp | **3.42** | **<0.0001** | 0.492 | 1.0000 | -4.48 | 0.4866 |
| Cha_pap-Cha_asp | -0.03 | 1.0000 | -0.258 | 1.0000 | -2.38 | 0.9940 |
| Cha_acu-Cha_asp | **1.33** | **0.0052** | -0.213 | 1.0000 | -0.71 | 0.9999 |
| Cha_tom-Cha_asp | -0.36 | 1.0000 | -0.192 | 1.0000 | 1.59 | 0.9757 |
| Cha_glo-Cha_asp | **4.87** | **<0.0001** | 0.290 | 1.0000 | -3.10 | 0.5153 |
| Nit_obt-Cha_asp | **1.41** | **0.0165** | 0.371 | 1.0000 | -4.63 | 0.0899 |
| Nit_fle-Cha_asp | **3.55** | **<0.0001** | 1.842 | 0.2668 | 4.39 | 0.3292 |
| Cha_vul-Nit_hya | 2.58 | 0.2831 | -0.350 | 1.0000 | -6.60 | 0.5099 |
| Cha_pap-Nit_hya | -0.87 | 1.0000 | -1.100 | 1.0000 | -4.50 | 0.9441 |
| Cha_acu-Nit_hya | 0.49 | 1.0000 | -1.055 | 0.5931 | -2.83 | 0.9819 |
| Cha_tom-Nit_hya | -1.20 | 1.0000 | -1.034 | 0.8694 | -0.53 | 1.0000 |
| Cha_glo-Nit_hya | **4.03** | **0.0107** | -0.552 | 1.0000 | -5.22 | 0.6454 |
| Nit_obt-Nit_hya | 0.57 | 1.0000 | -0.471 | 1.0000 | -6.75 | 0.3199 |
| Nit_fle-Nit_hya | 2.71 | 0.1681 | 1.000 | 1.0000 | 2.27 | 0.9973 |
| Cha_pap-Cha_vul | **-3.45** | **0.0102** | -0.750 | 1.0000 | 2.10 | 0.9989 |
| Cha_acu-Cha_vul | -2.08 | 0.1796 | -0.705 | 0.5657 | 3.77 | 0.6967 |
| Cha_tom-Cha_vul | **-3.78** | **<0.0001** | -0.684 | 0.8814 | 6.07 | 0.1429 |
| Cha_glo-Cha_vul | 1.45 | 1.0000 | -0.202 | 1.0000 | 1.38 | 0.9990 |
| Nit_obt-Cha_vul | -2.00 | 0.2817 | -0.121 | 0.9173 | -0.15 | 1.0000 |
| Nit_fle-Cha_vul | 0.14 | 1.0000 | 1.350 | 1.0000 | 8.87 | 0.0205 |
| Cha_acu-Cha_pap | 1.37 | 0.5645 | 0.045 | 1.0000 | 1.67 | 0.9995 |
| Cha_tom-Cha_pap | -0.33 | 1.0000 | 0.066 | 1.0000 | 3.97 | 0.8834 |
| Cha_glo-Cha_pap | **4.90** | **<0.0001** | 0.548 | 1.0000 | -0.72 | 1.0000 |
| Nit_obt-Cha_pap | 1.45 | 0.5784 | 0.629 | 1.0000 | -2.25 | 0.9959 |
| Nit_fle-Cha_pap | **3.58** | **0.0031** | 2.100 | 0.4365 | 6.77 | 0.4010 |
| Cha_tom-Cha_acu | **-1.69** | **0.0007** | 0.021 | 1.0000 | 2.30 | 0.8313 |
| Cha_glo-Cha_acu | **3.54** | **<0.0001** | 0.503 | 0.2150 | -2.39 | 0.8018 |
| Nit_obt-Cha_acu | 0.08 | 0.9607 | 0.584 | 0.0579 | -3.92 | 0.2260 |
| Nit_fle-Cha_acu | **2.22** | **0.0350** | **2.055** | **0.0061** | 5.10 | 0.1684 |
| Cha_glo-Cha_tom | **5.23** | **<0.0001** | 0.482 | 0.4711 | -4.69 | 0.0833 |
| Nit_obt-Cha_tom | **1.77** | **0.0027** | 0.563 | 0.1533 | **-6.22** | **0.0075** |
| Nit_fle-Cha_tom | **3.91** | **<0.0001** | **2.034** | **0.0157** | 2.80 | 0.8357 |
| Nit_obt-Cha_glo | **-3.46** | **0.0001** | 0.081 | 1.0000 | -1.53 | 0.9803 |
| Nit_fle-Cha_glo | -1.32 | 1.0000 | 1.552 | 1.0000 | **7.49** | **0.0090** |
| Nit_fle-Nit_obt | 2.14 | 0.0815 | 1.471 | 1.0000 | **9.02** | **0.0010** |

**SI4**: Mean, standard deviation (in round brackets) and coefficients of variation (in square brackets, calculated as standard deviation/mean*100) of leaf functional traits of the analyzed macrophyte groups. For leaf traits abbreviations see SI3 (LFW=Leaf Fresh Weight, LDW=Leaf Dry Weight). Sub.broad.leaf=Submerged broad-leaved species, Sub.narrow.leaf=Submerged narrow-leaved species.

| **Group** | **LFW**  [g] | **LDW**  [g] | **LA**  [mm^2^] | **LDMC**  [mg/g] | **SLA**  [mm^2^/mg] | **Chl-a**  [μg/g FW] | **Chl-b**  [μg/g FW] | **Car**  [μg/g FW] | **Chla/**  **Chlb** | **Chl/**  **Car** | **P**  [% DW] | **N**  [% DW] | **C**  [% DW] |
| --- | --- | --- | --- | --- | --- | --- | --- | --- | --- | --- | --- | --- | --- |
| Charophytes | 0.18 (0.18) [100.56] | 0.03 (0.03) [95.29] | 331.84 (248.34) [74.84] | 183.18 (82.67) [45.13] | 15.02 (11.66) [77.62] | 420.43 (248.47) [59.1] | 141.24 (86.32) [61.12] | 176.83 (113.01) [63.9] | 3.05 (0.29) [9.61] | 3.72 (2.13) [57.1] | 0.15 (0.08) [55.3] | 1.09 (0.59) [53.62] | 25.77 (3.59) [13.94] |
| Lemnids | 0.003 (0.003) [97.54] | 0.0002 (0.0002) [81.05] | 11.65 (8.77) [75.31] | 81.28 (26.79) [32.97] | 50.31 (18.51) [36.8] | 1134.53 (444.13) [39.15] | 370.34 (147.57) [39.85] | 323.81 (110.47) [34.11] | 3.14 (0.67) [21.47] | 4.62 (0.66) [14.22] | 0.53 (0.2) [37.81] | 4.07 (0.96) [23.47] | 38.09 (3.53) [9.26] |
| Nymphaeids | 126.63 (142.82) [112.79] | 16.9 (21.26) [125.81] | 122479 (147592) [120.5] | 123.17 (39.83) [32.34] | 9.43 (4.55) [48.29] | 915.68 (682.03) [74.48] | 232.76 (155.74) [66.91] | 290.57 (182.34) [62.75] | 3.86 (0.64) [16.58] | 4.05 (1.3) [32.14] | 0.31 (0.14) [43.78] | 3.06 (0.73) [24.02] | 42.66 (1.05) [2.46] |
| Sub.  Broad-leaf | 0.16 (0.09) [54.02] | 0.04 (0.02) [57.84] | 1022.16 (775.9) [75.91] | 221.96 (42.5) [19.15] | 35.67 (11.13) [31.19] | 1819.4 (1324.6) [72.81] | 597.84 (461.03) [77.12] | 507.44 (269.34) [53.08] | 3.16 (0.29) [9.07] | 4.41 (1.27) [28.9] | 0.17 (0.09) [52.06] | 2.84 (0.93) [32.71] | 39.57 (1.01) [2.54] |
| Sub.  Narrow-leaf | 0.01 (0.01) [89.64] | 0.001 (0.002) [119.94] | 45.24 (36.89) [81.54] | 117.76 (69.06) [58.65] | 50.27 (22.05) [43.87] | 1554.65 (874.28) [56.24] | 505.96 (304.38) [60.16] | 373.43 (172.03) [46.07] | 3.17 (0.35) [11.14] | 5.32 (1.19) [22.44] | 0.31 (0.22) [70.03] | 3.06 (1.1) [36.03] | 34.37 (5.59) [16.27] |
| Utricularids | 0.01 (0.01) [128.12] | 0.0006 (0.0008) [139.17] | 59.84 (83.84) [140.1] | 65.28 (20.19) [30.92] | 139.34 (54.23) [38.92] | 405.52 (166.34) [41.02] | 164.19 (69.74) [42.47] | 145.69 (70.11) [48.12] | 2.52 (0.61) [24.04] | 4.2 (1.08) [25.78] | 0.18 (0.06) [35.7] | 2.85 (1.16) [40.77] | 39.06 (1.34) [3.44] |

**SI5**: Results of the post hoc tests (Tukey – dark grey, or Dunn test – light grey) for the comparison of traits among macrophyte groups. Diff=mean difference between groups, P-adj= adjusted p-value. Significance level set at p<0.05, significant differences are highlighted in bold. For traits abbreviations see SI3.

|  | **log(LA)**  **[mm^2^]** | | **SLA**  **[mm^2^/mg]** | | **LDMC**  **[mg/g]** | |  |  |
| --- | --- | --- | --- | --- | --- | --- | --- | --- |
| **Comparison** | Diff | P-adj | Diff | P-adj | Diff | P-adj |  |  |
| Charophytes - Lemnids | **3.74** | **<0.0001** | **-35.28** | **<0.0001** | **101.90** | **<0.0001** |  |  |
| Charophytes - Nymphaeids | **-5.69** | **<0.0001** | **5.60** | **<0.0001** | **60.00** | **<0.0001** |  |  |
| Lemnids - Nymphaeids | **-9.43** | **<0.0001** | **40.88** | **<0.0001** | **-41.90** | **0.0001** |  |  |
| Charophytes - Sub.broad.leaf | **-0.71** | **0.0002** | **-20.65** | **<0.0001** | **-38.79** | **0.0027** |  |  |
| Lemnids - Sub.broad.leaf | **4.46** | **<0.0001** | 14.63 | 1.0000 | **-140.69** | **<0.0001** |  |  |
| Nymphaeids - Sub.broad.leaf | **4.97** | **<0.0001** | **-26.25** | **<0.0001** | **-98.79** | **<0.0001** |  |  |
| Charophytes - Sub.narrow.leaf | **2.01** | **<0.0001** | **-35.25** | **<0.0001** | **65.41** | **<0.0001** |  |  |
| Lemnids - Sub.narrow.leaf | **-1.73** | **<0.0001** | 0.04 | 0.8706 | **-36.49** | **0.0375** |  |  |
| Nymphaeids - Sub.narrow.leaf | **7.69** | **<0.0001** | **-40.84** | **<0.0001** | 5.41 | 0.2470 |  |  |
| Sub.broad.leaf - Sub.narrow.leaf | **2.72** | **<0.0001** | -14.60 | 1.0000 | **104.20** | **<0.0001** |  |  |
| Charophytes - Utricularids | **1.99** | **<0.0001** | **-124.32** | **<0.0001** | **117.90** | **<0.0001** |  |  |
| Lemnids - Utricularids | **-1.75** | **<0.0001** | **-89.03** | **0.0480** | 16.00 | 0.2074 |  |  |
| Nymphaeids - Utricularids | **7.67** | **<0.0001** | **-129.91** | **<0.0001** | **57.90** | **<0.0001** |  |  |
| Sub.broad.leaf - Utricularids | **2.70** | **<0.0001** | **-103.67** | **0.0003** | **156.69** | **<0.0001** |  |  |
| Sub.narrow.leaf - Utricularids | -0.02 | 1 | **-89.07** | **0.0043** | **52.49** | **<0.0001** |  |  |
|  |  |  |  |  |  |  |  |  |
|  | **Chl-a**  **[μg/g FW]** | | **Car**  **[μg/g FW]** | | **Chl-a/Chl-b** | | **Chl/Car** | |
| **Comparison** | Diff | P-adj | Diff | P-adj | Diff | P-adj | Diff | P-adj |
| Charophytes - Lemnids | **-714.10** | **<0.0001** | **-146.98** | **<0.0001** | -0.087 | 0.9581 | **-0.900** | **0.0400** |
| Charophytes - Nymphaeids | **-495.25** | **<0.0001** | **-113.74** | **<0.0001** | **-0.806** | **<0.0001** | -0.330 | 0.2703 |
| Lemnids - Nymphaeids | **218.85** | **0.0294** | 33.24 | 0.1180 | **-0.719** | **<0.0001** | 0.570 | 0.4117 |
| Charophytes - Sub.broad.leaf | **-1398.97** | **<0.0001** | **-330.61** | **<0.0001** | -0.111 | 0.7787 | -0.688 | 0.0706 |
| Lemnids - Sub.broad.leaf | -684.87 | 1.0000 | -183.63 | 0.3290 | -0.024 | 1.0000 | 0.212 | 0.9919 |
| Nymphaeids - Sub.broad.leaf | **-903.72** | **<0.0001** | **-216.87** | **<0.0001** | **0.695** | **<0.0001** | -0.358 | 0.6961 |
| Charophytes - Sub.narrow.leaf | **-1134.22** | **<0.0001** | **-196.59** | **<0.0001** | -0.119 | 0.4115 | **-1.595** | **<0.0001** |
| Lemnids - Sub.narrow.leaf | -420.12 | 1.0000 | -49.61 | 0.5850 | -0.031 | 0.9997 | -0.695 | 0.2530 |
| Nymphaeids - Sub.narrow.leaf | **-638.97** | **<0.0001** | **-82.86** | **<0.0001** | **0.688** | **<0.0001** | **-1.265** | **<0.0001** |
| Sub.broad.leaf - Sub.narrow.leaf | 264.75 | 0.9347 | 134.02 | 0.1823 | -0.008 | 1.0000 | -0.907 | 0.0100 |
| Charophytes - Utricularids | 14.91 | 1.0000 | 31.14 | 0.2568 | **0.526** | **<0.0001** | -0.473 | 0.1379 |
| Lemnids - Utricularids | **729.02** | **<0.0001** | **178.12** | **<0.0001** | **0.613** | **<0.0001** | 0.427 | 0.7735 |
| Nymphaeids - Utricularids | **510.16** | **<0.0001** | **144.88** | **<0.0001** | **1.332** | **<0.0001** | -0.143 | 0.9723 |
| Sub.broad.leaf - Utricularids | **1413.88** | **<0.0001** | **361.75** | **<0.0001** | **0.637** | **<0.0001** | 0.215 | 0.9693 |
| Sub.narrow.leaf - Utricularids | **1149.13** | **<0.0001** | **227.73** | **<0.0001** | **0.645** | **<0.0001** | **1.122** | **<0.0001** |

|  | **P**  **[% DW]** | | **N**  **[% DW]** | | **C**  **[% DW]** | |  |  |
| --- | --- | --- | --- | --- | --- | --- | --- | --- |
| **Comparison** | Diff | P-adj | Diff | P-adj | Diff | P-adj |  |  |
| Charophytes - Lemnids | **-0.378** | **<0.0001** | **-2.98** | **<0.0001** | **-12.31** | **<0.0001** |  |  |
| Charophytes - Nymphaeids | **-0.158** | **<0.0001** | **-1.96** | **<0.0001** | **-16.89** | **<0.0001** |  |  |
| Lemnids - Nymphaeids | **0.221** | **<0.0001** | **1.02** | **<0.0001** | **-4.58** | **<0.0001** |  |  |
| Charophytes - Sub.broad.leaf | -0.021 | 0.9880 | **-1.75** | **0.0012** | **-13.79** | **<0.0001** |  |  |
| Lemnids - Sub.broad.leaf | **0.357** | **<0.0001** | 1.23 | 0.0657 | -1.48 | 0.8941 |  |  |
| Nymphaeids - Sub.broad.leaf | **0.137** | **<0.0001** | 0.22 | 0.9958 | 3.10 | 0.1832 |  |  |
| Charophytes - Sub.narrow.leaf | **-0.157** | **<0.0001** | **-1.97** | **<0.0001** | **-8.60** | **<0.0001** |  |  |
| Lemnids - Sub.narrow.leaf | **0.221** | **<0.0001** | **1.02** | **0.0011** | **3.72** | **0.0001** |  |  |
| Nymphaeids - Sub.narrow.leaf | 0.001 | 1.0000 | 0.00 | 1.0000 | **8.29** | **<0.0001** |  |  |
| Sub.broad.leaf - Sub.narrow.leaf | **-0.136** | **<0.0001** | -0.22 | 0.9966 | **5.20** | **0.0036** |  |  |
| Charophytes - Utricularids | -0.024 | 0.9714 | **-1.75** | **<0.0001** | **-13.29** | **<0.0001** |  |  |
| Lemnids - Utricularids | **0.354** | **<0.0001** | **1.23** | **<0.0001** | -0.98 | 0.6870 |  |  |
| Nymphaeids - Utricularids | **0.134** | **<0.0001** | 0.21 | 0.8052 | **3.60** | **<0.0001** |  |  |
| Sub.broad.leaf - Utricularids | -0.003 | 1.0000 | -0.01 | 1.0000 | 0.50 | 0.9990 |  |  |
| Sub.narrow.leaf - Utricularids | **0.133** | **<0.0001** | 0.21 | 0.9303 | **-4.69** | **<0.0001** |  |  |

**SI6:** Correlation matrix of leaf functional traits of all macrophyte groups included in the niche comparison analysis. Pearson’s correlation coefficients are shown on the lower panels, while data points are shown on the upper panels. For traits abbreviations see SI3. LA, LDMC, SLA, Chl-*a*, Chl-*a*/Chl-*b*, Chl/Car, P and C were included in the analysis.

**SI7**: Complete PCA biplots visualizing the three PC axes used for the development of the hypervolumes. Variance explained by each axis is indicated in brackets. For traits abbreviations see SI3.

**SI8**: Credible intervals of relative niche volume, uniqueness and intersection, obtained with bootstrap permuting each niche 499 times. Relative volume is the volume of each group divided by the union of all groups volumes. Relative uniqueness is the unshared volume of each group divided by its total volume. Relative intersection is the volume shared by a pair of groups divided by the union of all groups volume. Q 2.5=quantile 2.5, Q 97.5=quantile 97.5. For relative volume and uniqueness, non-overlapping intervals indicate significant difference between groups at the 0.05 level. For relative intersection, intervals excluding zero indicate significant overlap between groups at the 0.05 level.

| **Metric** | **Group** | **mean** | **Q 2.5** | **Q 97.5** |
| --- | --- | --- | --- | --- |
| **Relative volume** | Charophytes | 0.1694 | 0.1460 | 0.1947 |
|  | Lemnids | 0.1471 | 0.0793 | 0.2175 |
|  | Nymphaeids | 0.2254 | 0.1947 | 0.2577 |
|  | Sub.broad.leaf | 0.1110 | 0.0551 | 0.1757 |
|  | Sub.narrow.leaf | 0.4907 | 0.4256 | 0.5537 |
|  | Utricularids | 0.1148 | 0.0935 | 0.1364 |
| **Relative uniqueness** | Charophytes | 0.4170 | 0.3108 | 0.5309 |
|  | Lemnids | 0.2454 | 0.1247 | 0.3436 |
|  | Nymphaeids | 0.9163 | 0.8801 | 0.9506 |
|  | Sub.broad.leaf | 0.7477 | 0.6553 | 0.8434 |
|  | Sub.narrow.leaf | 0.5416 | 0.4521 | 0.6368 |
|  | Utricularids | 0.8405 | 0.7643 | 0.9467 |
| **Relative intersection** | Charophytes - Lemnids | 0.0363 | 0.0171 | 0.0529 |
|  | Charophytes - Nymphaeids | 0.0000 | 0.0000 | 0.0000 |
|  | Charophytes - Sub.broad.leaf | 0.0114 | 0.0023 | 0.0221 |
|  | Charophytes - Sub.narrow.leaf | 0.1706 | 0.1379 | 0.1996 |
|  | Charophytes - Utricularids | 0.0028 | 0.0000 | 0.0073 |
|  | Lemnids - Nymphaeids | 0.0476 | 0.0249 | 0.0686 |
|  | Lemnids - Sub.broad.leaf | 0.0052 | 0.0000 | 0.0176 |
|  | Lemnids - Sub.narrow.leaf | 0.1775 | 0.0989 | 0.2416 |
|  | Lemnids - Utricularids | 0.0020 | 0.0000 | 0.0075 |
|  | Nymphaeids - Sub.broad.leaf | 0.0003 | 0.0000 | 0.0023 |
|  | Nymphaeids - Sub.narrow.leaf | 0.0089 | 0.0039 | 0.0142 |
|  | Nymphaeids - Utricularids | 0.0000 | 0.0000 | 0.0000 |
|  | Sub.broad.leaf - Sub.narrow.leaf | 0.0461 | 0.0195 | 0.0789 |
|  | Sub.broad.leaf - Utricularids | 0.0000 | 0.0000 | 0.0000 |
|  | Sub.narrow.leaf - Utricularids | 0.0307 | 0.0102 | 0.0459 |
